# Trends in Tuberculosis — United States, 2012

**Published:** 2013-03-22

**Authors:** Roque Miramontes, Robert Pratt, Sandy F. Price, Thomas R. Navin, Terrence Q. Lo

**Affiliations:** Div of TB Elimination, National Center for HIV/AIDS, Viral Hepatitis, STD, and TB Prevention; EIS Officer, CDC

In 2012, a total of 9,951 new tuberculosis (TB) cases were reported in the United States, an incidence of 3.2 cases per 100,000 population. This represents a decrease of 6.1% from the incidence reported in 2011 and is the 20th consecutive year of declining rates. Of the 3,143 counties in the United States, 1,388 (44.2%) did not report a new TB case during 2010–2012. This report summarizes provisional TB surveillance data reported to CDC’s National Tuberculosis Surveillance System in 2012. The TB rate in foreign-born persons in the United States was 11.5 times as high as in U.S.-born persons. In comparison with non-Hispanic whites, TB rates among non-Hispanic Asians, Hispanics, and non-Hispanic blacks were 25.0, 6.6, and 7.3 times as high, respectively. Although the number of cases dropped below 10,000 for the first time since standardized national reporting of TB began in 1953, a number of challenges remain that slow progress toward the goal of TB elimination in the United States. Initiatives to increase TB awareness and testing and treatment of latent infection and disease will be critical to TB elimination efforts, especially among foreign-born populations, racial/ethnic minorities, and other groups that are disproportionately affected.

Health departments in the 50 states and the District of Columbia electronically report to CDC verified TB cases that meet the CDC and Council of State and Territorial Epidemiologists surveillance case definition.* Reports include the patient’s county of residence, self-identified race and ethnicity (i.e., Hispanic or non-Hispanic), human immunodeficiency virus (HIV) status, drug-susceptibility test results, country of origin, and information on excessive alcohol use, homelessness, and detention at a correctional facility. CDC calculates national and state TB rates overall and by racial/ethnic group, using U.S. Census Bureau population estimates (1). The Current Population Survey provides the population denominators used to calculate TB rates and percentage changes according to national origin.† For TB surveillance, a U.S.-born person is defined as a person born in the United States or its associated jurisdictions,§ or a person born in a foreign country but having at least one U.S.-citizen parent. In 2012, the country of birth was unknown for 0.4% of patients and race/ethnicity was unknown for 0.9%. In this report, persons of Hispanic ethnicity might be of any race; non-Hispanic persons are categorized as Asian, black, white, American Indian/Alaska Native, Native Hawaiian or other Pacific Islander, or of multiple races.

In 2012, a total of 9,951 new tuberculosis (TB) cases were reported in the United States. The incidence of 3.2 cases per 100,000 population was a decrease of 6.1% from the incidence reported in 2011 (Table) and is the 20th consecutive year of declining rates. Although reported TB cases and rates decreased from 2011 for foreign-born and U.S.-born persons and among all racial/ethnic groups, foreign-born persons and racial/ethnic minorities continued to be disproportionately affected by TB in the United States. The TB rate among foreign-born persons in the United States in 2012 was 11.5 times higher than among U.S.-born persons. The TB rates among Asians, Hispanics, and blacks were 25.0, 6.6, and 7.3 times as high as whites, respectively. Among U.S.-born persons, the greatest disparity in TB rates was between blacks and whites; the rate among blacks was 5.8 times as high as that of whites.

Individual state TB rates per 100,000 population varied widely, from 0.4 in West Virginia to 9.0 in Alaska (median: 2.3). Rates in 2012 were lower than in 2011 in 33 states and the District of Columbia and higher in 17 states. Four states (California, Texas, New York, and Florida) each reported more than 500 cases for 2012, as they have since 2008. Combined, these four states accounted for 4,967 TB cases, representing half (49.9%) of all TB cases reported in 2012. Among the 441 counties in these four states, 136 (30.8%) did not report a new TB case during 2010–2012. Among the 2,702 counties in the states reporting fewer than 500 cases, 1,253 (46.4%) counties did not report a TB case during 2010–2012 (Figure 1).

Among U.S.-born persons, the number and rate of TB cases decreased in 2012. The 3,666 TB cases reported among U.S.-born persons (37.0% of all cases with known national origin) represented an 8.2% decline compared with 2011 and a 57.6% decline compared with 2000 (Figure 2). The rate of 1.4 per 100,000 population among U.S.-born persons represents a 8.7% decline since 2011 and a 61.4% decline since 2000.

Among foreign-born persons in the United States, the number and rate of TB cases also decreased in 2012. A total of 6,243 TB cases were reported among foreign-born persons (63.0% of all cases in persons with known national origin), a 4.1% decline since 2011 and an 18.1% decline since 2000. The 15.8 cases per 100,000 population TB rate among foreign-born persons represents an 8.6% decline since 2011 and 42.3% decline since 2000. In 2012, 54.6% of foreign-born persons with TB and known country of birth originated from five countries: 1,303 (20.9%) from Mexico, 768 (12.3%) from the Philippines, 529 (8.5%) from India, 450 (7.2%) from Vietnam, and 351 (5.6%) from China.

Asians had the highest TB case rate among all racial/ethnic groups, which was 25.0 times higher than that of whites (Table). From 2011 to 2012, TB rates per 100,000 population decreased most for Hispanics (9.1%), followed by blacks (7.4%), Asians (5.6%), and whites (5.2%). Among persons in the United States with TB and known national origin, 95.7% of Asians, 75.0% of Hispanics, 39.7% of blacks, and 19.1% of whites were foreign-born. Among U.S.-born persons with TB, blacks were the racial/ethnic group most affected (36.7%). The TB rate among U.S.-born blacks was 5.8 times greater than for U.S.-born whites, the largest disparity among U.S.-born persons.

In 2012, HIV status was known for >80% of TB cases reported. Among those with a known result, 7.7% were reported as HIV-positive.

Among persons aged ≥15 years with TB and known housing status, 5.6% reported being homeless within the past year. Among persons aged ≥15 years, 12.1% reported excessive alcohol use within the past year. Among persons aged ≥15 years and known status, 4.2% were confined to a correctional facility (i.e., prison, jail, or juvenile correctional facility) at the time of TB diagnosis.

A total of 127 cases of multidrug-resistant TB (MDR TB)¶ were reported in 2011, the most recent year for which complete drug-susceptibility results are available. Drug-susceptibility test results for isoniazid and rifampin were reported for 97.0% and 96.8% of culture-confirmed TB cases in 2010 and 2011, respectively. Among these cases, the percentage of MDR TB for 2011 (1.6% [127 of 7,817 cases]) was greater than the percentage for 2010 (1.3% [109 of 8,241 cases]). The percentage of MDR TB cases among persons without a previous history of TB was 1.3% in 2011. For persons with a previous history of TB, the percentage with MDR TB was 8.2% in 2011. Foreign-born persons accounted for 109 (85.8%) of the 127 MDR TB cases in 2011. One case of extensively drug-resistant TB** has been reported for 2012.

## Editorial Note

Since the resurgence of TB in the late 1980s and early 1990s, when TB cases increased substantially, the United States has experienced 20 consecutive years of declines in TB cases and rates. If TB rates had remained constant at their 1993 level, more than 200,000 additional TB cases would have occurred in the United States during 1993–2012 (Michael P. Chen, PhD, CDC, personal communication, 2013).†† Instead, substantial federal, state, and local resources have been mobilized to strengthen TB control efforts.§§ Most areas of the country report fewer cases of TB, and no TB cases have been reported for the past 3 years in 44.2% of counties. However, TB cases among foreign-born persons, persons infected with HIV, homeless persons, those who are incarcerated, and those who report excessive alcohol use are significant challenges that impede progress toward TB elimination. Drug-resistant TB also is a global public health issue that has the potential to affect a greater proportion of U.S. TB cases (2).

Geographically, the distribution of TB cases is heterogeneous. Although all states and the District of Columbia reported cases of TB in their jurisdictions, four states (California, Texas, New York, and Florida) reported half of all TB cases in the United States. These four states have less than one third of the U.S. population. Rates of TB were highest in Alaska (9.0 per 100,000 population) and Hawaii (8.4 per 100,000), which combined have <1% of the U.S. population. Additionally, 17 states had higher rates of TB in 2012 than in 2011. At the state level, the distribution of TB by county also is heterogeneous.

TB persists in specific populations. In 2012, foreign-born persons and racial/ethnic minorities continued to be affected disproportionately. Although the numbers and rates of TB among foreign-born persons in the United States decreased, they did so at a lower rate than for U.S.-born persons. Asians continued to be the racial/ethnic group most represented among new TB cases. Initiatives that promote further TB awareness, testing, and treatment of latent infection and TB disease among foreign-born persons and racial/ethnic minorities will be critical for future TB elimination efforts.

Homeless persons also are a population at high risk for TB (3). Persons who are homeless might have factors that favor TB transmission, such as excessive alcohol use, substance abuse, malnutrition, and crowded living situations, as reported in recent outbreaks in the United States (4,5). Vigilance for TB among homeless persons will be crucial for maintaining progress toward TB elimination among the U.S.-born population.

The findings in this report are subject to at least two limitations. First, this analysis is limited to reporting provisional TB cases and case rates for 2012. Second, case rates are calculated from estimates, not counts, of population denominators from 2012. Final TB case rates based on updated denominators will be presented in CDC’s annual TB surveillance report later this year.

What is already known on this topic?

As tuberculosis (TB) has declined in the United States since 1993, an increasing proportion of cases have been among foreign-born persons. Among U.S.-born persons with TB, racial and ethnic minorities are affected disproportionately.

What is added by this report?

In 2012, the number of TB cases reported in the United States was 9,951, the lowest number since standardized national reporting of TB began in 1953. The incidence decreased to 3.2 cases per 100,000 population from 3.4 in 2011, the 20th consecutive annual decrease. Most cases were among foreign-born persons, in whom the incidence was 11.5 times higher than among U.S.-born persons. Rates also varied by race/ethnicity, with rates 25.0, 6.6, and 7.3 times as high in non-Hispanic Asians, Hispanics, and non-Hispanic blacks as in non-Hispanic whites, respectively.

What are the implications for public health practice?

Continued vigilance and surveillance of TB is needed to reach CDC’s TB elimination goal of <1 case per 1 million persons. Initiatives to improve awareness, testing, and treatment of TB disease and latent TB infection in foreign-born and minority populations are likely to be the most efficient way to facilitate progress toward the elimination of TB in the United States.

Despite the decline in TB cases and rates from the previous year, the rate of 3.2 TB cases per 100,000 persons in 2012 exceeds CDC’s TB elimination goal for 2010 of <1 case per 1 million population (6). Although continued progress toward achieving TB elimination in the United States occurred in 2012, TB persists in some geographic regions and among foreign-born persons and racial/ethnic minorities. Counties that reported no TB cases in 2010–2012 still require TB prevention and control activities because TB can cross borders. Ongoing surveillance will be essential to shape targeted TB prevention strategies in the effort to sustain success toward TB elimination in the United States.

## Figures and Tables

**FIGURE 1:**
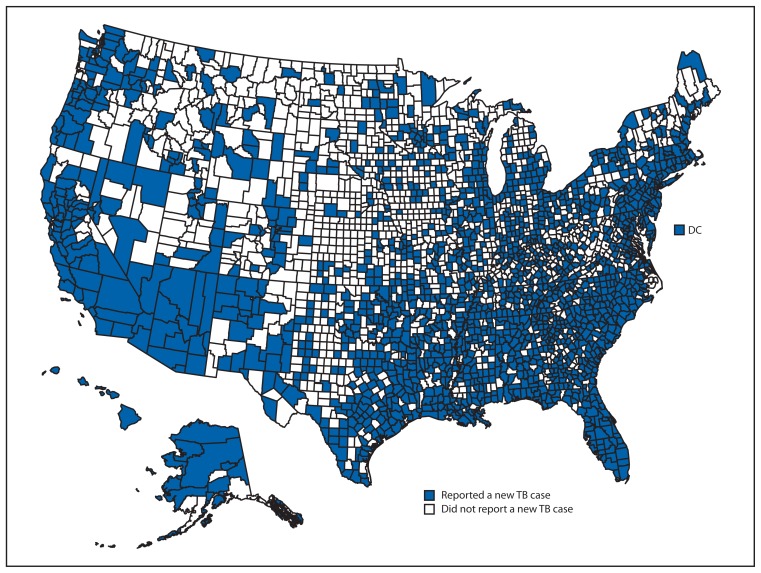
Reported new tuberculosis (TB) cases, by county — United States, National Tuberculosis Surveillance System, 2010–2012^†^ ^†^ Data are current as of March 6, 2013. Data for 2012 are provisional.

**FIGURE 2:**
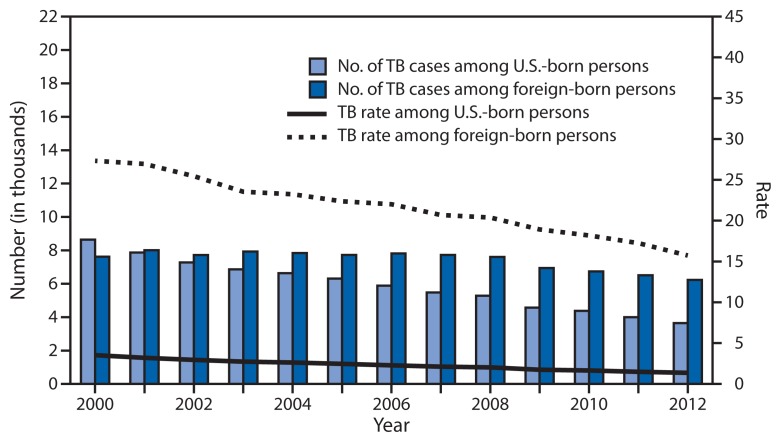
Number and rate* of tuberculosis (TB) cases among U.S.-born and foreign-born persons, by year reported — United States, National Tuberculosis Surveillance System, 2000–2012^†^ * Per 100,000 population. ^†^ Data current as of February 15, 2013. Data for 2012 are provisional.

**TABLE:** Number and rate* of tuberculosis cases and percentage change, by race/ethnicity — United States, National Tuberculosis Surveillance System, 2011–2012†

	2011		2012		% change 2011–2012		U.S. population	
				
Race/Ethnicity	No.	Rate	No.	Rate	No.	Rate	2011	2012
Asian	3,156	21.0	3,043	19.8	−3.6	−5.6	15,063,596	15,382,833
Black	2,379	6.2	2,222	5.7	−6.6	−7.4	38,337,168	38,664,702
White	1,650	0.8	1,566	0.8	−5.1	−5.2	197,510,927	197,638,915
Other§	248	2.9	240	2.7	−3.2	−5.7	8,634,949	8,859,335
Hispanic	2,999	5.8	2,793	5.2	−6.9	−9.1	52,045,277	53,305,237
Unknown	92	—	87	—				
**Total**	**10,524**	**3.4**	**9,951**	**3.2**	**−5.4**	**−6.1**	**311,591,917**	**313,851,022**

* Per 100,000 population.

† Data as of February 22, 2013. Data for 2012 are provisional.

§ Includes American Indian/Alaska Native (2011: n = 128, rate = 5.6 per 100,000 population; 2012: n = 139, rate = 6.0), Native Hawaiian or other Pacific Islander (2011: n = 82, rate = 16.1; 2012: n = 67, rate = 12.9), and multiple race (2011: n = 38, rate = 0.7; 2012: n = 34, rate = 0.6).

## Footnotes

* Available at http://wwwn.cdc.gov/nndss/script/casedef.aspx?condyrid=876&datepub=1/1/2009%2012:00:00%20am.

† Additional information available http://dataferrett.census.gov.

§ Includes Guam, the Commonwealth of Northern Mariana Islands, America Samoa, the Federated States of Micronesia, Palau, and the Republic of the Marshall Islands.

¶ Defined by the World Health Organization as a case of TB in a person with a *Mycobacterium tuberculosis* isolate resistant to at least isoniazid and rifampin. Additional information available at http://whqlibdoc.who.int/publications/2010/9789241599191_eng.pdf.

** Defined by the World Health Organization as a case of TB in a person an *M. tuberculosis* isolate with resistance to at least isoniazid and rifampin among first-line anti-TB drugs, resistance to any fluoroquinolone (e.g., ciprofloaxacin or ofloxacin), and resistance to at least one second-line injectable drug (e.g., amikacin, capreomycin, or kanamycin). Additional information available at http://whqlibdoc.who.int/publications/2010/9789241599191_eng.pdf.

†† The unpublished analysis estimated the number of additional TB cases during 1993–2012 by two methods. The first method assumes the TB rate did not change from 1993 (a rate of 9.7 TB cases per 100,000), multiplies that rate by the annual estimated U.S. population to calculate the expected annual number of TB cases during 1993–2012, and sums the differences between the actual number of TB cases reported from the expected annually. The second method estimated the number of additional TB cases from two segmented log-linear models that assume the TB rate remained unchanged from 1993 and sums the differences between the actual number of TB cases reported from the expected cases as predicted by the models for each year. Both methods yielded >200,000 additional TB cases.

§§ Based on justification of estimates for Congressional Appropriations Committees for multiple years. Available at http://www.cdc.gov/fmo/topic/budget%20Information/index.html.
